# Mitochondrial Genomes of Three Species of the Family Camaenidae (Gastropoda: Stylommatophora): Structural Features, Codon Usage Patterns, and Phylogenetic Implications

**DOI:** 10.1002/ece3.72282

**Published:** 2025-10-12

**Authors:** Xing‐Ming Ran, Hai‐Yong He, Lin Yang, Jian‐Kun Long, Zhi‐Min Chang, Zhong Li, Junyi Gao, Xiang‐Sheng Chen

**Affiliations:** ^1^ Guizhou Key Laboratory of Agricultural Biosecurity Guizhou University Guiyang Guizhou P. R. China; ^2^ Institute of Entomology Guizhou University Guiyang Guizhou P. R. China; ^3^ The Provincial Special Key Laboratory for Development and Utilization of Insect Resources Guizhou University Guiyang Guizhou P. R. China; ^4^ Institute of Plant Protection Guizhou Academy of Agricultural Sciences Guiyang Guizhou P. R. China; ^5^ Guizhou Provincial Tobacco Company Bijie City Company Bijie Guizhou P. R. China

**Keywords:** Camaenidae, codon usage bias, phylogenetic analysis

## Abstract

This study sequenced the complete mitochondrial genomes of three Camaenidae snail species (*Acusta ravida*, *Bradybaena similaris* , and *Trichobradybaena submissa*). Each mitogenome contained the typical 37 genes (13 PCGs, 22 tRNAs, and 2 rRNAs), with most tRNAs forming clover structures (except trnK, trnH, trnN, trnS1, trnS2, and trnR). Comparative analysis with nine existing Camaenidae mitogenomes revealed: (1) The nucleotide composition of mitochondrial genes was significantly skewed toward A and T; (2) Most protein genes used ATN start codons and TAA/T‐ stop codons; (3) Synonymous codons ending with T/A were dominant. Analyses (PR2‐plot, neutrality plot) indicated codon usage was influenced by both mutation pressure and natural selection. Phylogenetically, the three newly sequenced species clustered within Bradybaeninae of Camaenidae, consistent with previous classifications. The results support Hygromiidae and Geomitridae as sister groups and identify Polygyridae as the sister clade to Camaenidae. Additionally, they also partially support elevating Satsuma and its subgenus to subfamily status. This study enriches mitogenomic resources for Camaenidae and provides foundational data for future research on codon usage, adaptive evolution, and phylogenetics within this family.

## Graphical abstract

(1) The complete mitochondrial genomes of three species within the family Camaenidae were sequenced and annotated; (2) Mitochondrial characteristics and codon usage patterns of the Camaenidae genome were compared and analyzed; (3) The phylogenetic relationship of Camaenidae was reconstructed.

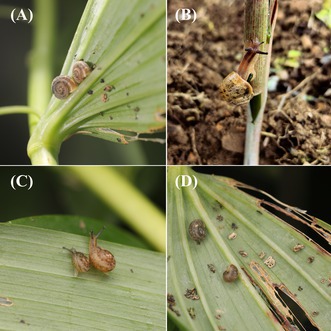

## 1 Introduction

**FIGURE 1:**
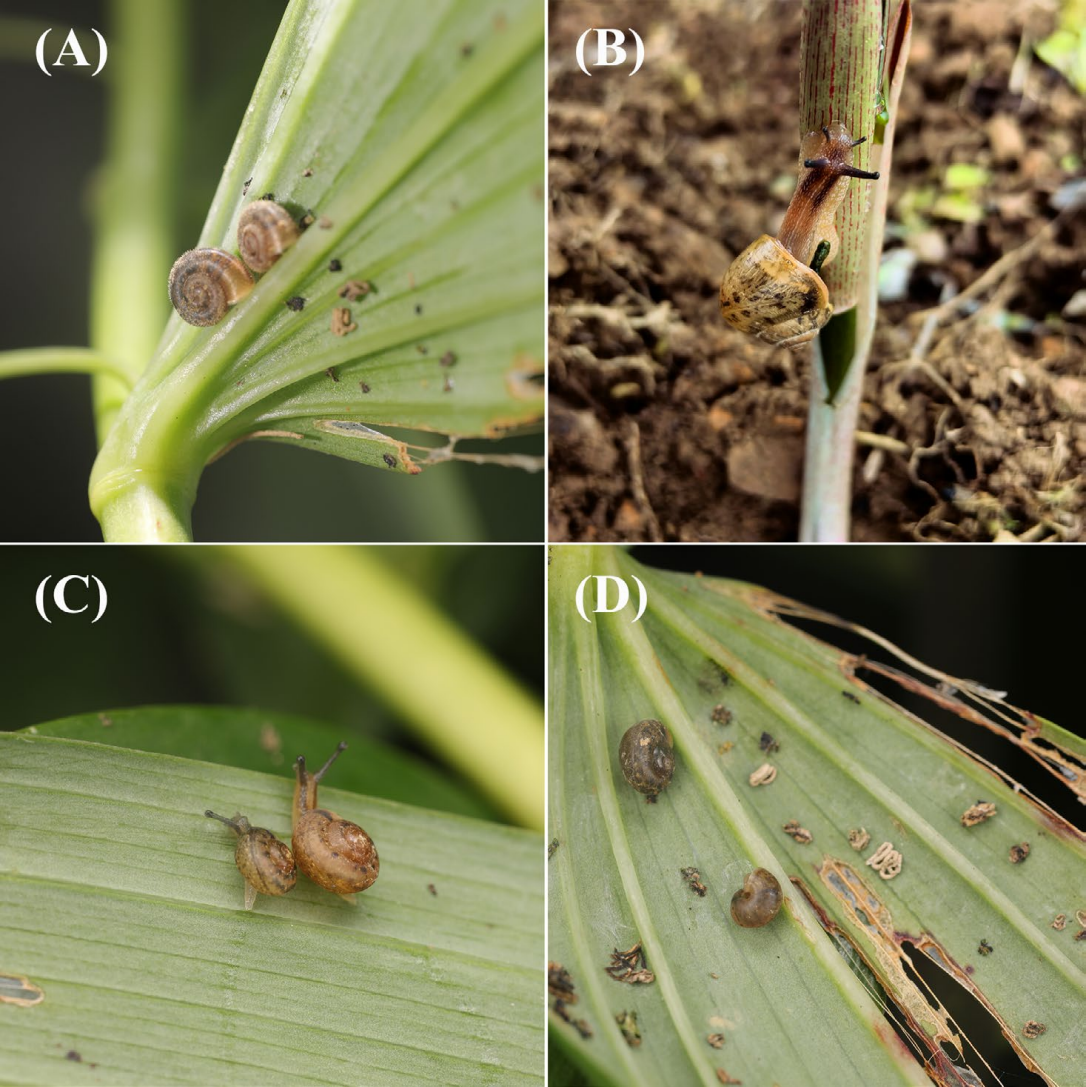
Ecological photographs of Polygonatum cyrtonema damage by three snail species. These snails feed on leaves, leaving fecal deposits, which in severe cases can induce disease and decay. (A) *Trichobradybaena submissa*, (B) *Acusta ravida*, (C) *Bradybaena similaris* , and (D) Polygonatum cyrtonema leaf damaged by snails.

The Camaenidae, a species‐rich gastropod family within the order Stylommatophora (Gastropoda: Stylommatophora) and predominantly phytophagous and saprophagous, exhibiting significant phytophagous impacts on agricultural crops, horticultural species, and silvicultural systems (Wang et al. 2014). Their herbivorous activities induce marked reductions in both crop yield and product quality, establishing this taxon as a significant agricultural pest (Radwan and Gad 2021). The three snail species in this study—*Acusta ravida* (Benson, 1842), *Bradybaena similaris* (Férussac, 1822), and *Trichobradybaena submissa* (Deshayes, 1874) cause severe damage to *Polygonatum cyrtonema* (Figure 1). Their stem feeding results in plant lodging, whereas secreted mucus and deposited stringy excrement contaminate leaves. Under suitable temperatures, these secretions can further promote disease development, leading to plant necrosis and decay.

Subsequent to Camaenidae's initial taxonomic delineation, the classification of this family has undergone successive revisions, including challenges to its monophyly and persistent issues in classifying its lower‐level taxa (Tillier 1989; Emberton and Tillier 1995; Wade et al. 2001; Cuezzo 2003; Bouchet and Rocroi 2005; Wade et al. 2006; Wade et al. 2007). Bouchet et al.'s (2017) revision of the family Camaenidae was based on previous phylogenetic studies—those relying on morphological characters and limited gene fragments. They merged the formerly recognized families Bradybaenidae and Camaenidae into a single family and propose that extant members primarily comprise the Asian and Australian subfamilies Camaeninae, Bradybaeninae, Hadrinae, and Helicostylinae. Although this revision resolved certain taxonomic conflicts, phylogenetic relationships within Camaenidae remain debated, largely stemming from early taxonomic approaches that relied heavily on unstable morphological characteristics (Wu 2019; Sutcharit et al. 2020; Chen et al. 2021; Páll‐Gergely et al. 2022; Zhang et al. 2024; Zhang and Wang 2025). For instance, Satsuma, previously classified under the subfamily Bradybaeninae of the family Camaenidae, has recently been proposed to be elevated to the rank of a distinct subfamily (Nordsieck 2017; Sei et al. 2017; Zhang et al. 2024). These uncertainties urgently require validation through novel tools such as mitochondrial genome sequencing.

The mitochondrial genome (mtDNA) typically comprises 37 functional regions (Boore 1999). The mtDNA possesses inherent strengths over nuclear DNA for evolutionary research, including: its small size, high structural conservation, and rapid mutation rate, making it widely applicable for resolving phylogenetic relationships, clarifying taxonomic disputes, and investigating population genetics (Cameron 2014; Ladoukakis and Zouros 2017), such as some cnidarians (Feng et al. 2023), fishes (Sun et al. 2025), gastropods (Inäbnit et al. 2025), and insects (Guo et al. 2024). Secondly, complete mtDNA sequences integrate richer phylogenetic signals, significantly enhancing the resolution and accuracy of species classification and phylogenetic inference (Powell et al. 2013; Li et al. 2022).

Moreover, the mtDNA also contains information such as Codon Usage Bias (CUB), the phenomenon of unequal usage frequency among synonymous codons encoding the same amino acids (Parvathy et al. 2022). It is closely related to gene expression, translation, and the overall function of proteins (Liu 2020). Analysis of CUB can provide a reference for further understanding of species' genetic evolution, environmental adaptation, and gene expression regulation (Deb et al. 2020). Genomic constraints (e.g., GC content), mutation burden gradients, directional selection pressures, and amino acid physicochemical profiles have been established as determinants of CUB (Deb et al. 2021).

The advent of high‐throughput DNA sequencing and advanced data mining, combined with plummeting sequencing costs, has driven a transformative shift in mitogenomic approaches. Consequently, mitochondrial genome sequencing has proliferated, furnishing robust frameworks for elucidating phylogenies and codon usage bias patterns. But current research on complete mitogenomes within the family Camaenidae is limited, with only nine reported prior to this study. This study sequenced mitogenomes of three Camaenidae species, combining multi‐strategy analysis of codon usage bias (CUB) and reconstructing phylogenies with 13 PCGs and two rRNAs, establishing foundations for evolutionary studies of Camaenidae and related taxa.

## 2 Materials and Methods

### 2.1 Specimen Collection, DNA Extraction and Sequencing

*A. ravida* , *B. similaris* , and *T. submissa* were collected from a *Polygonatum cyrtonema* cultivation base in Zunyi, Guizhou (May 2023), where they caused significant crop damage. For each species, five adult individuals were collected for mitochondrial genome sequencing. Specimens were fixed in anhydrous ethanol and stored at −40°C. No specific permits were required for the collection of these land snail species, as they are common pests and not endangered or protected in China. DNA was extracted from soft tissues using the TIANamp Kit (TIANGEN) according to the manufacturer's protocol.

DNA quality was assessed by 1% agarose gel electrophoresis to check for degradation and contamination, and concentration was measured using the Qubit DNA Assay Kit on a Qubit 2.0 Fluorometer (Life Technologies, CA, USA). All samples had a concentration ≥ 60 ng/μL and total DNA ≥ 3600 ng.

Sequencing libraries were constructed from 1 μg of DNA per sample using the CLEANNGS DNA kit, with unique index codes for each sample. Genomic DNA was fragmented to ~350 bp, end‐repaired, A‐tailed, and ligated with Illumina adapters. After PCR amplification, libraries were purified with the AMPure XP system and assessed for size distribution using an Agilent 2100 Bioanalyzer. Quantification was performed via real‐time PCR (3 nM).

Libraries were clustered on a cBot Cluster Generation System with NovaSeq 6000 S4 reagents and sequenced on the Illumina NovaSeq 6000 platform to generate 150 bp paired‐end reads. Each sample yielded > 7 GB of raw data. Voucher specimens and DNA samples are deposited at the Institute of Entomology, Guizhou University, Guiyang, China (GUGC), under voucher codes (CXS‐RXM20230501).

### 2.2 Mitochondrial Genome Assembly and Annotation

Illumina raw reads were quality‐checked using FastQC (v0.6; www.bioinformatics.babraham.ac.uk/projects/fastqc/) and trimmed with Trimmomatic (v0.33) to remove low‐quality bases on the basis of FastQC results. Putative mitogenome reads with an average quality value of < Q30 were removed before assembly. Mitochondrial genome assembly was performed using NOVOPlasty (Dierckxsens et al. 2016). The assembled data were preliminarily annotated using the MITOS Web Server (https://usegalaxy.eu/) (Bernt et al. 2013), on the basis of the mitochondrial genetic code of invertebrates, to determine the genomic positional ranges of genes. These annotations were then imported into Geneious Prime 2023 (Kearse et al. 2012). To ensure accurate annotation, especially for transfer and ribosomal RNA genes, the protein‐coding gene (PCG) boundaries were manually corrected and verified by aligning them with multiple published mitogenomes from closely related species within the Camaenidae family, including Aegista aubryana (GenBank: NC_029419), Mastigeulota_kiangsinensis (GenBank: NC_024935), and Dolicheulota_formosensis (GenBank: NC_027493). tRNA secondary structures were inferred using the MITOS Web Server (https://usegalaxy.eu/) with default parameters and covariance models. Finally, genome visualization was performed using Proksee (https://proksee.ca/) (Grant et al. 2023).

### 2.3 Nucleotide Composition and Diversity Analysis

Nucleotide composition analysis was performed in MEGA X (Kumar et al. 2018), including whole mitogenome base composition (A/T/C/G%); GC/AT skew; GC/AT content; the proportion of base types at the third position codons (A3/T3/C3/G3%) and GC12 values. Polymorphic sites and nucleotide diversity (Pi) for PCGs and rRNAs across 12 Camaenidae species (Table S1) were determined with DnaSP (Librado and Rozas 2009). A sliding window analysis (200‐bp window, 20‐bp step) was used to calculate Pi across PCGs and rRNAs.

### 2.4 Analysis of Codon Usage

RSCU values for mitochondrial PCGs were computed in MEGA X (Kumar et al. 2018), with corresponding heatmaps visualized using TBtools (Chen et al. 2023). Codon representation is defined as overrepresented (RSCU > 1.6) or underrepresented (RSCU < 0.6) at established thresholds. An RSCU value of 1.0 denotes unbiased usage, whereas values < 1.0 and > 1.0 indicate low and high preference, respectively (Deb et al. 2021).

### 2.5 Parity Rule2 (PR2) Bias Plot

The PR2 bias plot evaluates the relative contributions of mutation pressure and natural selection pressure to CUB. Theoretically, at point (0.5, 0.5), CUB remains unaffected by either force; under the theoretical scenario where gene composition is solely determined by base composition, A/T and G/C usage at third codon positions should occur with equal probability, whereas the combined effects of selective pressure and mutation pressure may lead to differential frequencies of A/T or G/C usage (Sueoka 1995).

### 2.6 Neutrality Plot

Neutrality plots were constructed to quantify the relative influence of natural selection and mutation pressure on mitochondrial PCGs codon usage bias. Pearson correlation analysis between GC12 and GC3 was performed in SPSS. A regression slope approaching 0 (with non‐significant correlation) indicates CUB is primarily governed by natural selection. Conversely, a slope approaching 1 (with significant correlation) reflects the dominance of mutation pressure (Sueoka 1988).

### 2.7 Phylogenetic Analysis

All genomic data utilized for phylogenetic analyses are accessible via GenBank. Three closely related species (*Arion vulgaris* GenBank: NC_046044, *Meghimatium bilineatum* GenBank: NC_024935, *Lissachatina fulica* GenBank: NC_024601) were used as the outgroup for the analysis (Table S1).

Nucleotide sequences of the 13 PCGs and 2 rRNAs were extracted, aligned, trimmed, and concatenated using PhyloSuite (Zhang et al. 2020). Initial MAFFT alignments (Nakamura et al. 2018) were enhanced with MACSE codon optimization (Ranwez et al. 2018) to address MAFFT's inability to model codon boundary architecture in PCGs. Final trimming used GBlocks for PCGs (Talavera and Castresana 2007) and trimAI for rRNAs (Capella‐Gutiérrez et al. 2009), followed by concatenation of polished alignments into partitioned datasets.

PartitionFinder (Lanfear et al. 2016) selected optimal substitution models for partitioned datasets. Phylogenies were reconstructed using: (1) Maximum Likelihood (ML): IQ‐tree (Nguyen et al. 2015) with 10,000 ultrafast bootstrap replicates; (2) Bayesian Inference (BI): MrBayes (Huelsenbeck and Ronquist 2001) run for 2,000,000 generations (sampled every 1000 generations; 25% burn‐in), with convergence confirmed when the average standard deviation of split frequencies (ASDSF) stabilized at ≤ 0.01.

## 3 Results

### 3.1 Nucleotide Composition and Diversity Analysis

**FIGURE 2:**
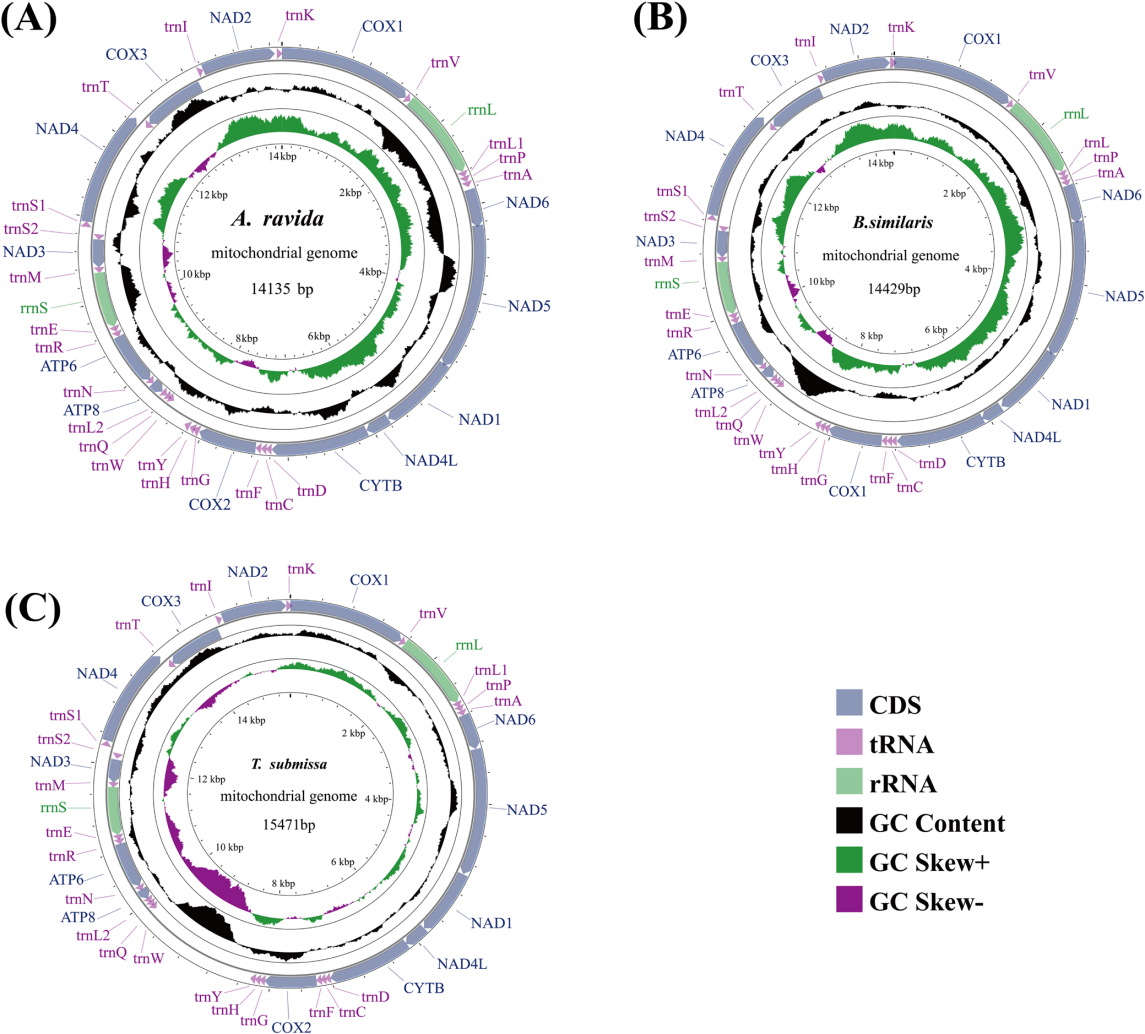
Mitochondrial genome maps of three species of A (A. ravida), B (B. similaris), and C (T. submissa). The chain is marked with an arrow indicating the direction of gene transcription. Gene lengths correspond to nucleotide lengths in the diagram. The outermost layer is the H‐chain, and the second layer is the L‐chain.

**FIGURE 3:**
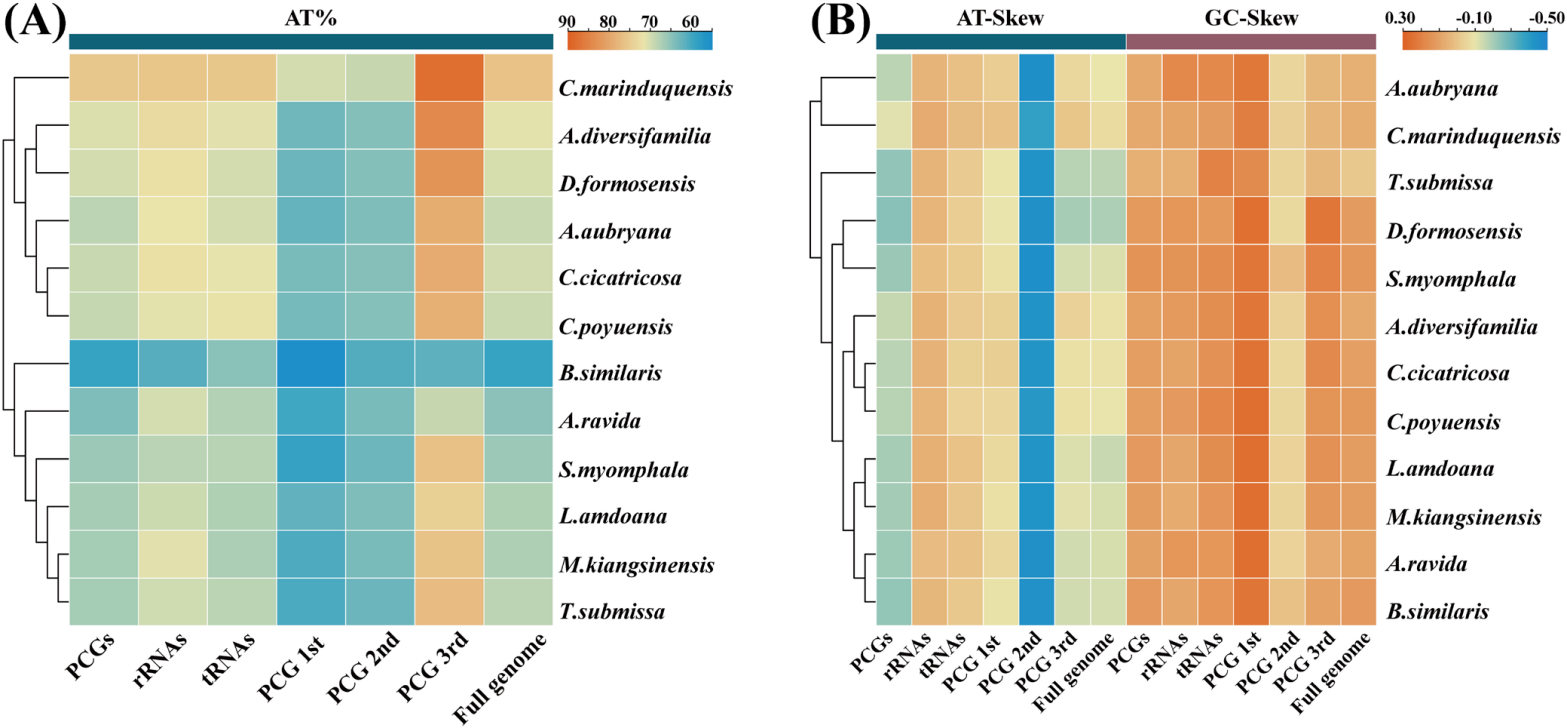
Nucleotide composition of various datasets of Camaenidae mitogenomes. Hierarchical clustering of Camaenidae species (*y*‐axis) based on (A) the A + T content; (B) the AT‐skew and GC‐skew.

The complete mitogenomes of *A. ravida* (14,135 bp; GenBank PQ166711), *B. similaris* (14,429 bp; PQ180368), and *T. submissa* (15,471 bp; PQ180369) each contain 37 genes (13 PCGs, 22 tRNAs, two rRNAs) with conserved strand asymmetry: 9 PCGs + rrnL + 13 tRNAs on the heavy (H) strand versus 4 PCGs + rrnS + 9 tRNAs on the light (L) strand (Figure 2). Comparative analysis of these three new and nine published Camaenidae mitogenomes revealed size variation from 13,798 bp to 15,471 bp, where *T. submissa* represents the largest recorded mitogenome in this family. All 12 species exhibit significant AT bias (59.2% in *B. similaris* to 76.3% in *C. marinduquensis*) and consistently negative AT skew (−0.191 to −0.067). Notably, *T. submissa* uniquely shows negative GC‐skew (−0.007), contrasting with positive GC‐skew values (0.062–0.133) in other species (Figure 3).

**FIGURE 4:**
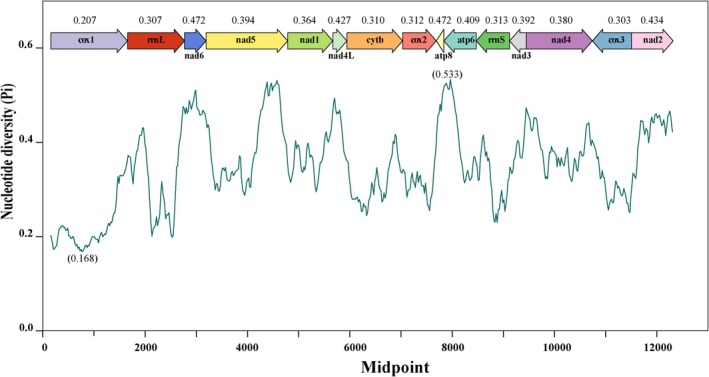
Sliding window analysis presenting the distribution of Pi values across PCGs and rRNA genes, as evaluated in mitochondrial genomes of 12 Camaenidae species. Window length: 100 bp; step size: 20.

Sliding‐window analysis of 12 Camaenidae mitogenomes revealed significant nucleotide diversity (Pi) variation across 13 PCGs and two rRNAs (Figure 4). The most polymorphic region occurred within ATP6 (Pi = 0.533), whereas the most conserved segment was located in COX1 (Pi = 0.168). Among individual genes, ATP8 and NAD6 (both Pi = 0.472) showed the highest variability, contrasting with COX1 (Pi = 0.207) as the most conserved PCG. Both rRNAs exhibited moderate conservation (rrnS Pi = 0.307; rrnL Pi = 0.313).

### 3.2 tRNA and rRNA

Comparative analyses of the 12 Camaenidae species showed that the total length of tRNA ranges from 1281 bp to 1396 bp, with an AT content ranging from 65% to 75.8%, AT skew from −0.041 to 0.024, and GC skew from 0.122 to 0.2; the total length of rRNA ranges from 1671 bp to 1741 bp, with a gene AT content varying from 61.2% to 75.9%, exhibiting positive AT skew (0.02–0.076) and GC skew (0.058–0.183) (Figure 3). In addition, the tRNA secondary structures of the three species tested were predicted. Although structural prediction confirmed the classic cloverleaf conformation for virtually all tRNA genes across the three new mitogenomes (Figures S1–S3), specific tRNAs exhibited structural anomalies: (i) A. *ravida* : trnK lacked the acceptor stem; trnH and trnN were deficient in TψC arms; trnS1 and trnS2 lacked DHU arms. (ii) B. *similaris* : trnN lacked the TψC arm, whereas trnR, trnS1, and trnS2 were missing DHU arms. (iii) T. *submissa*: trnS2 exhibited TψC arm loss and trnS1 lacked the DHU arm. Seven types of non‐Watson‐Crick base pairings were identified (G‐U, U–U, U‐C, A‐C, A‐G, A‐A, and G‐G), with GU mismatches being the most frequent. Notably, mismatched A‐A and G‐G pairs each occurred once in *T. submissa*—specifically in trnN and trnS1, respectively.

### 3.3 PCGs and Codon Usage

**FIGURE 5:**
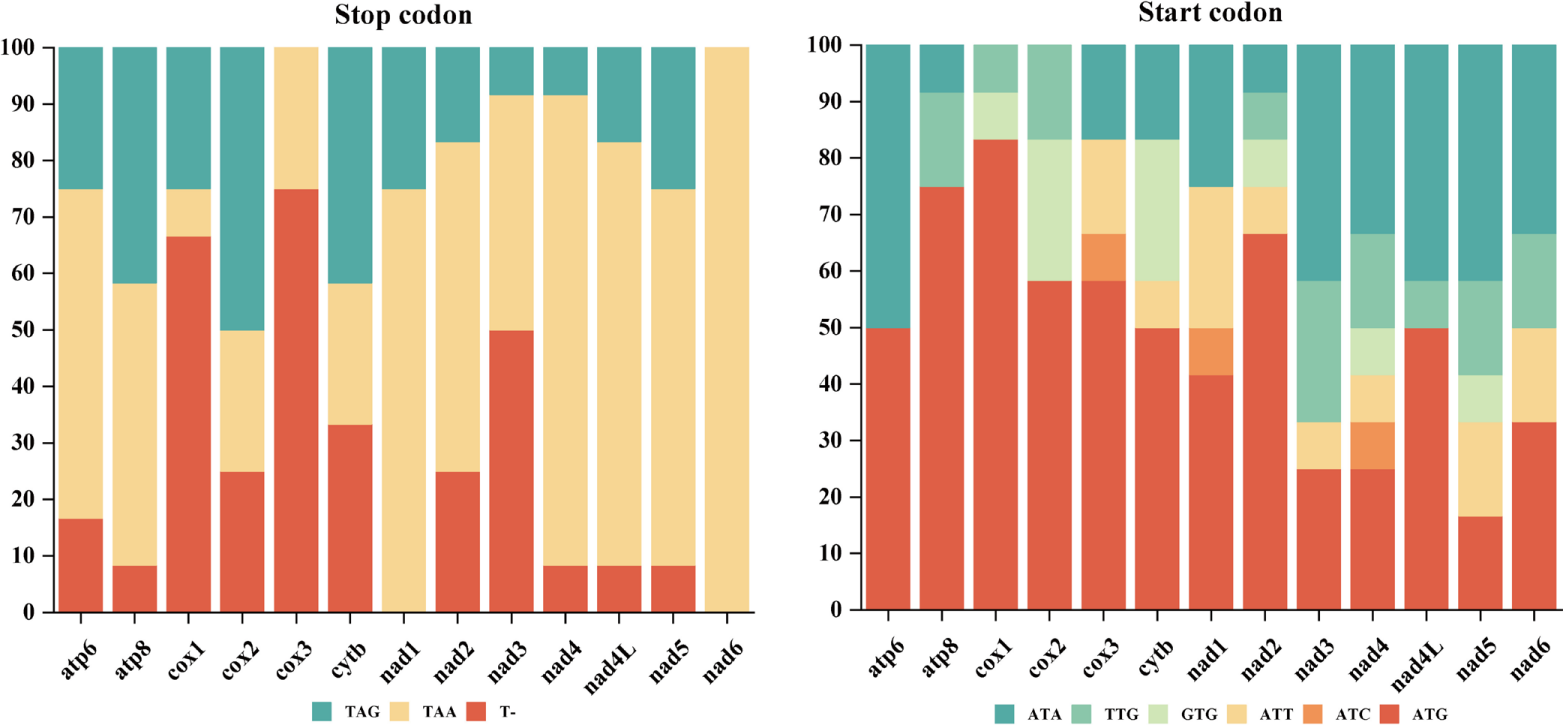
The frequency distribution of start codons and stop codons of 13 PCGs in Camaenidaes.

**FIGURE 6:**
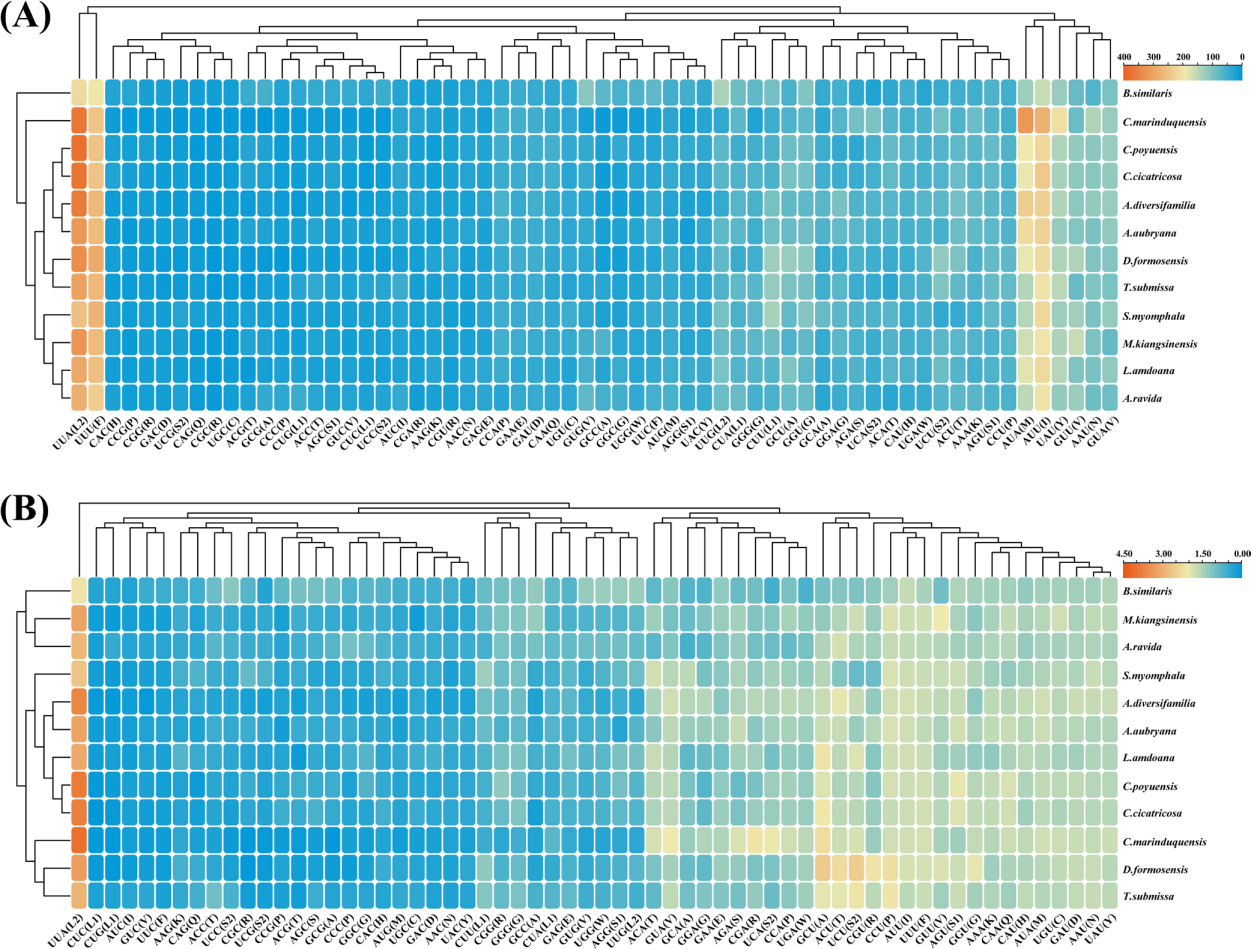
The codon number (A) and RSCU values (B) per codon in the mitochondrial PCGs of 12 Camaenidae species.

Across all 12 Camaenidae mitogenomes, the 13 PCGs collectively span 10,692–10,992 bp with AT content 59.2%–75.7%, AT skew −0.243 to −0.112, and GC skew 0.061–0.150 (Figure 3). Most PCGs initiate with standard ATN codons (predominantly ATG), whereas a minority utilize TTG and GTG as alternative start codons; termination predominantly uses TAA, with TAG and incomplete T‐ also observed (Figure 5). Figure 6 presents codon usage and RSCU values for PCGs in the studied Camaenidae mitogenomes. Analysis revealed a pronounced AT bias, with high‐frequency codons exhibiting AT‐rich composition: UUA (mean frequency: 319), UUU (256), and AUU (218). Conversely, underrepresented codons showed GC enrichment, exemplified by CAG (11), UGC (10), and CGC (7). RSCU analysis of 62 non‐termination codons demonstrated significant usage imbalance: 10 codons were overrepresented (RSCU > 1.6) and 23 underrepresented (RSCU < 0.6). Notably, the most frequent codons universally terminate in A or U nucleotides.

### 3.4 Parity Rule2 (PR2) Bias Plot Analysis

**FIGURE 7:**
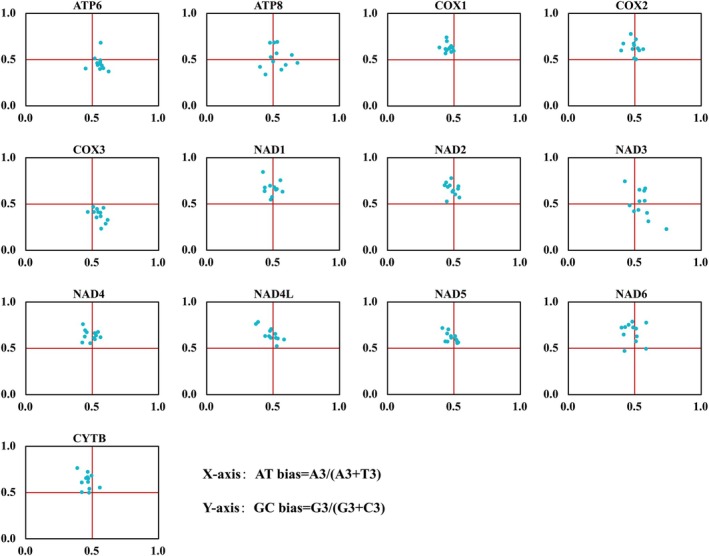
PR2‐plot analysis of 13 PCGs, where each point represents one of the 12 evaluated species.

Compositional relationships among third‐codon‐position nucleotides in all 13 PCGs are visualized through PR2‐plot analysis in Figure 7. Eight PCGs (CYTB, COX1, COX2, NAD1, NAD2, NAD4, NAD4L, and NAD5) clustered within quadrants I and II, indicating higher G versus C usage frequency at third codon positions. The remaining PCGs showed off‐center distribution, reflecting unequal base frequencies (A/T/G/C) at their synonymous sites. These results demonstrate that codon preferences in Camaenidae mitogenomes are influenced by both mutation pressure and natural selection.

### 3.5 Neutrality Plot Analysis

**FIGURE 8:**
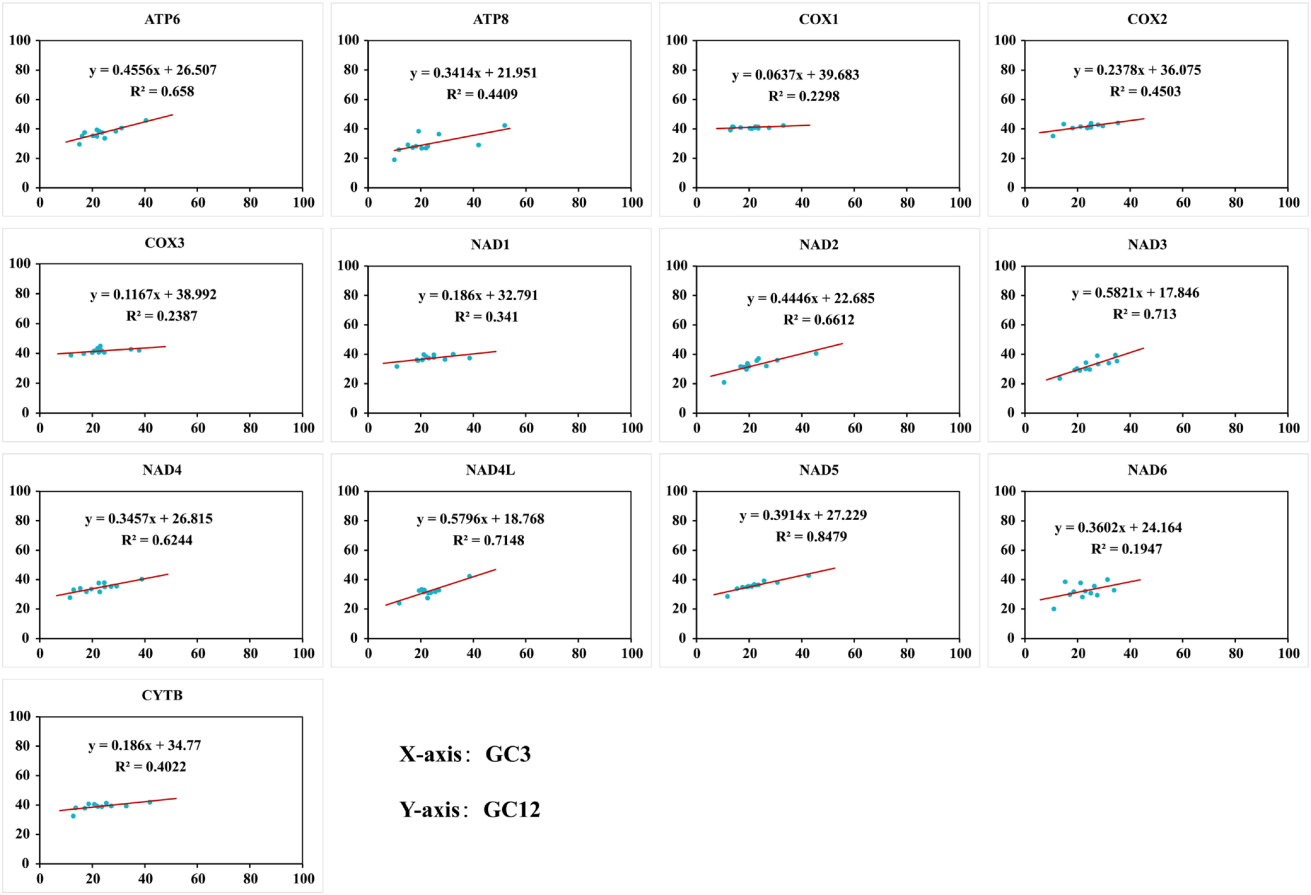
Neutrality plot analysis of 13 PCGs showing the correlation between GC values in the first, second, and third codon positions in the 12 evaluated species.

Figure 8 illustrates the neutral plot analysis outcomes for all 13 PCGs. GC12 vs. GC3 correlation analysis (Table S2) shows significant positive relationships in 10 protein‐coding genes (*p* < 0.01 for ATP6, ND2, ND3, ND4, ND4L, ND5; *p* < 0.05 for ATP8, COX2, CYTB, and ND1), demonstrating that mutation pressure governs CUB in these genes. Non‐significant correlations in the remaining PCGs indicate predominant selection pressure influence. Furthermore, regression slopes below 0.5 in 11 of 13 mitochondrial genes (excluding ND3 and ND4L) confirm natural selection as a major CUB determinant. Consequently, combined evidence establishes that ATP6, ATP8, COX2, CYTB, ND1, ND2, ND4L, and ND5 experience co‐dominant effects from both mutation pressure and natural selection, whereas ND3 and ND4L are primarily mutation‐driven. Finally, the remaining two genes are predominantly shaped by natural selection.

### 3.6 Phylogenetic Analysis

**FIGURE 9:**
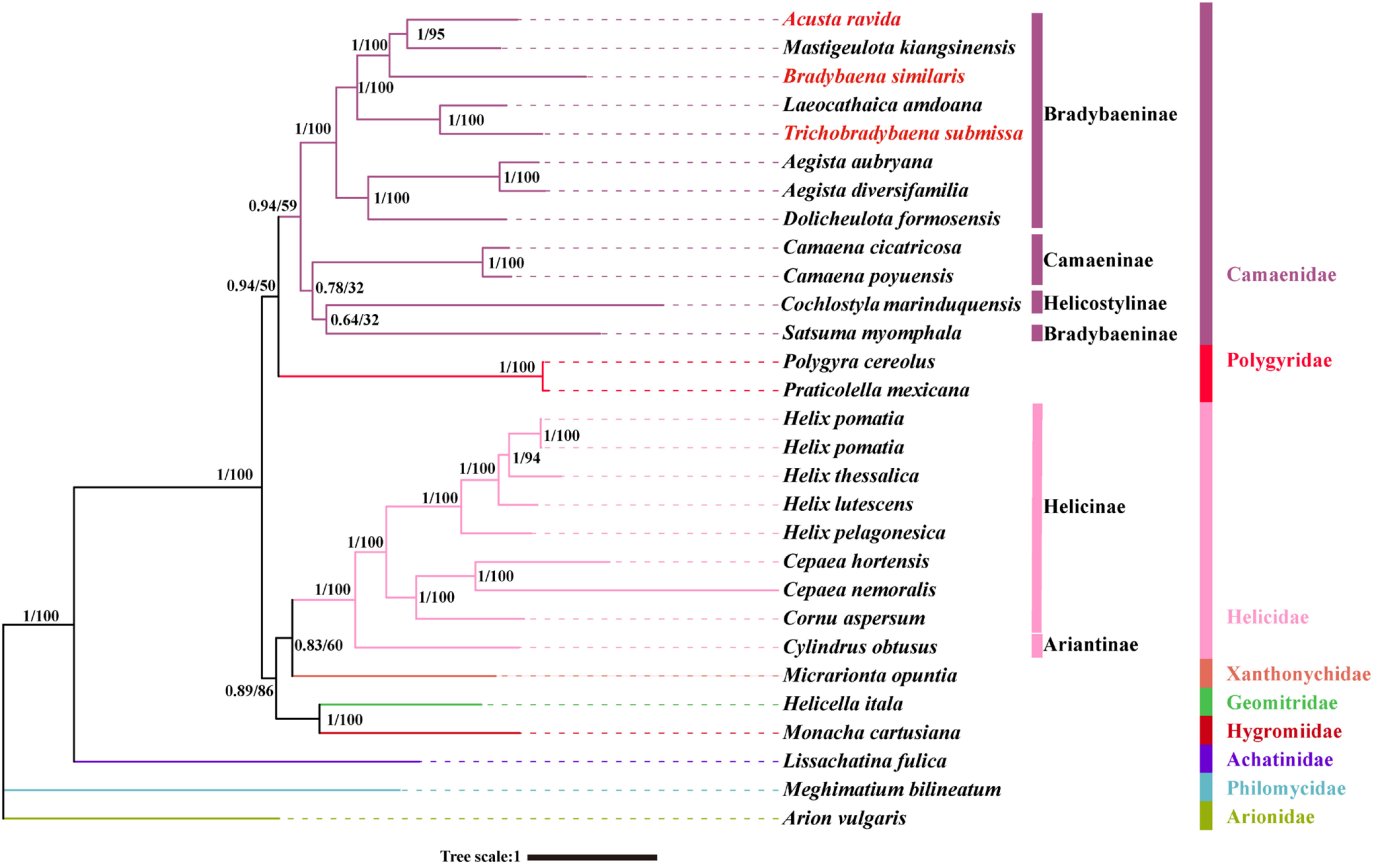
Phylogenetic tree inferred by maximum likelihood (ML) and maximum parsimony (MP) methods based on 13 protein genes and 2 rRNAs. The numbers on the branches indicate the posterior probabilities (BI/ML).

Phylogenetic analysis of mitogenomes revealed identical genus‐level topologies between ML trees and BI trees, and most nodes received high support values (Figure 9). Newly sequenced three species were clustered into Bradybaeninae within Camaenidae. The entire superfamily Helicoidea was divided into two clades. The first clade (Helicidae + Xanthonychidae) + (Geomitridae + Hygromiidae) was well‐supported (PP = 0.89, BP = 86). Helicidae is a monophyletic clade with maximum support values (PP = 1.00, BP = 100). The family Xanthonychidae (represented by *Micrarionta opuntia* ) forms a sister group to Helicidae, with strong support in BI (PP = 0.83) but only moderate support in ML (BP = 60). Geomitridae, represented by *Helicella itala*, and Hygromiinae, represented by *Monacha cartusiana*, were confirmed as sister taxa with full support (PP = 1, BP = 100).

The second clade, consisting of Camaenidae and Polygyridae, received strong support in BI (PP = 0.93), but weaker support in ML (BP = 50). In this branch, the monophyly of Polygyridae, represented by *Polygyra cereolus* and *Praticolella mexicana*, received strong support (PP = 1.00, BP = 100). Within the Camaenidae clade, Helicostylinae (represented by *Cochlostyla marinduquensis*) clustered with *Satsuma myomphala* (PP = 0.64, BP = 32) and formed a sister group with Camaeninae (PP = 0.78, BP = 32). In contrast, the remaining Bradybaeninae (excluding *Satsuma myomphala*) were fully supported as a monophyletic group (PP = 1.00, BP = 100).

## 4 Discussion

### 4.1 Mitochondrial Genome Characteristics of Camaenidae

The three newly sequenced Camaenidae mitogenomes (14,135–15,471 bp) contain 37 canonical genes (13 PCGs, 22 tRNAs, 2 rRNAs). Consistent with most gastropod taxa, their gene expression profiles and structural organization conform to the classical mitochondrial gene hypothesis (Wang et al. 2014; Shen and Wu 2023; Inäbnit et al. 2025; Wang et al. 2025). Comparative analysis revealed intra‐family size variation (13,798–15,471 bp), with *T. submissa* possessing the longest sequence. These differences are primarily attributed to the variable‐sized non‐coding regions (Grande et al. 2008). Mitochondrial genomes of Camaenidae exhibit a pronounced AT bias, with nucleotide composition strongly favoring adenine and thymine. This pattern aligns with findings across other Stylommatophora species (Yang et al. 2019). Such elevated AT content corresponds to the hallmark characteristics of Stylommatophora, which demonstrate the highest AT values among pulmonate gastropods (Boore 1999; Gaitán‐Espitia et al. 2013).

In the Camaenidae, nucleotide diversity analysis highlights COX1 as the most conserved gene with minimal evolutionary change, whereas ATP8 and NAD6 exhibit the highest variability with accelerated evolutionary rates. This analytical approach is widely employed to identify regions of elevated nucleotide divergence, providing critical guidance for selecting species‐specific markers, particularly in taxa with high morphological similarity (Ma et al. 2020; Huang et al. 2023). To some extent, these highly divergent genes may serve as potential molecular markers for species delimitation and population genetics (Yang et al. 2021; Guo et al. 2022).

### 4.2 Domain Loss and Base Mismatches in tRNAs

Structural anomalies in tRNAs, including absent TΨC or DHU arms, incomplete acceptor stems, and non‐Watson‐Crick base pairs, were identified in the mitogenomes of *A. ravida* , *B. similaris* , and *T. sumissa*. The loss of these structures is common among metazoans, including some mollusks, and is believed to result from selective pressure to reduce mitochondrial genome size (Yamazaki et al. 1997; Boore 1999; Masta 2000; Guzmán et al. 2021). However, the conservation of these genes implies the existence of robust compensatory mechanisms to maintain translational function.

Although these deviations can potentially impair key processes such as aminoacylation, ribosome binding, and codon recognition, their functionality is often preserved through molecular adaptations. For example, a report about nematode mitochondria showed that an evolved extra elongation factor EF‐Tu could assist with the interaction between truncated tRNAs and conventional EF‐Tu, thereby enabling their normal function (Watanabe et al. 2014; An et al. 2023).

The most common mismatch, G‐U, forms stable wobble pairs that are functionally accepted in ribosomal decoding. Although the base geometry of G‐U pairs results in weaker bonds compared to A‐U pairs, they are still capable of forming hydrogen bonds and are relatively stable (Golden et al. 2020). Consequently, G‐U mismatches typically function as “quasi‐normal pairings” and are ubiquitously observed in nearly all mitochondrial tRNAs (Varani and McClain 2000). More non‐canonical mismatches (e.g., U–U, A‐A, and A‐C) may be corrected by RNA editing, a process demonstrated in land snails that restores canonical base‐pairing, particularly in the acceptor stem (Yokobori and Pääbo 1995). Additionally, extensive post‐transcriptional nucleotide modifications can stabilize aberrant structures and fine‐tune decoding efficiency, thereby mitigating the negative impacts of genomic simplification (Berg and Brandl 2021).

Thus, the persistence of structurally abnormal tRNAs reflects an evolutionary trade‐off between genome minimization and functional integrity. Compensatory mechanisms like RNA editing and tRNA modification offset these structural defects, enabling the production of functional tRNAs from a minimized genome (D'Souza and Minczuk 2018). This genomic plasticity may represent an adaptive strategy in snails, potentially facilitating rapid response to environmental pressures, such as those encountered by pest species adapting to new hosts or habitats by tolerating molecular‐level imperfection in favor of evolutionary flexibility (Yokobori and Pääbo 1995).

### 4.3 Codon Usage Patterns in Camaenidae

The 13 PCGs across 12 mitogenomes predominantly initiate with ATN start codons (ATA/T/G/C), supplemented by minor TTG/GTG usage. Termination primarily involved TAA/TAG and incomplete T‐codons. This genomic pattern is well‐documented across metazoans, including mollusks (Wang et al. 2014; Zhu et al. 2023; Zhao et al. 2023; Guo et al. 2024; Long et al. 2024; Liang et al. 2024). Within this framework, the incomplete stop codon T‐ is postulated to undergo functional restoration via post‐transcriptional polyadenylation (Ojala et al. 1981).

The codon usage exhibits a pronounced AT bias, with AT‐rich codons being favored over GC‐rich counterparts. This bias is evidenced by RSCU values demonstrating non‐random utilization within synonymous codon families, as codons containing A or T at the third position are preferentially selected. The codon capture theory suggests that the extreme deviation of genomic GC/AT content has a strong impact on codon usage and may even lead to the disappearance of some codons (Santos et al. 2004). This variation plays critical roles in modulating protein folding dynamics, functional regulation, and translational efficiency (Quax et al. 2015).

Codon usage patterns are fundamentally governed by the interplay of mutational biases and selective pressures (Sharp et al. 2010; Wang et al. 2018). Our PR2 and neutrality plot analyses demonstrate that codon usage bias (CUB) in Camaenidae mitogenomes is shaped by a complex interplay of mutation pressure and natural selection, with the relative strength of these forces varying across protein‐coding genes (PCGs) (Figures 7 and 8; Table S2). A strong mutational signature was observed in ND3 and ND4L, as evidenced by high GC12‐GC3 correlation and regression slopes exceeding 0.5. This pronounced mutation pressure may be linked to the specific structural context of the mitogenome or localized base composition fluctuations. In contrast, natural selection predominates in core respiratory genes such as COX1 and CYTB, likely driven by strong purifying selection to optimize translational efficiency and protein function for oxidative phosphorylation (OXPHOS).

This gene‐specific variation underscores the tension between genome‐level mutational constraints and functional optimization. Mechanistically, mutation pressure, reflecting the AT/GC bias inherent in replication and repair processes, provides the foundational sequence variation. Natural selection, potentially mediated by translational efficiency tied to mitochondrial tRNA abundance, then acts upon this variation to favor optimal codons that enhance translational accuracy and speed (Nath Choudhury et al. 2017; Liu 2020). The pervasive off‐center distribution in PR2 plots supports the widespread action of such selective fine‐tuning across most genes.

The co‐dominance of both forces in genes like ATP6 and ND1 indicates an evolutionary equilibrium where selection works within the constraints of the mutational landscape. This mutation‐selection interaction pattern has been increasingly recognized in the mitochondrial genomes of various other taxa, including mollusks (Sun and Gao 2017; Gu et al. 2025). Future studies integrating mitochondrial tRNA gene copy number and expression data will be critical to precisely elucidate the mechanisms of translational selection in mollusks.

### 4.4 Phylogenetic Inference

On the basis of a concatenated dataset of 13 protein‐coding genes (PCGs) and two ribosomal RNAs (rRNAs), this study reconstructed the phylogenetic framework of the superfamily Helicoidea. Although maximum likelihood (ML) and Bayesian inference (BI) produced congruent topologies at the genus level and above, several deep nodes exhibited notable discrepancies in support values (Figure 9). Inconsistencies in phylogenetic support, such as the sister relationship between Xanthonychidae and Helicidae (BI PP = 0.83 vs. ML BP = 60) and the clade containing Camaenidae and Polygyridae (BI PP = 0.93 vs. ML BP = 50), may arise from multiple factors. These include heterogeneity in evolutionary rates among data partitions, inherent differences between Bayesian posterior probabilities and ML bootstrap proportions, and potential model misspecification. Therefore, although mitochondrial genomic data provide a robust overall framework, they may be insufficient alone to resolve phylogenetic relationships among closely related families within the superfamily.

Despite the aforementioned uncertainties, the results of this study yield strong phylogenetic signals in multiple aspects and align with mainstream views. In our phylogenetic reconstruction, the three newly sequenced species clustered within the subfamily Bradybaeninae of the family Camaenidae. The subfamily Ariantinae (represented by Cylindrus obtusus) formed a distinct clade separate from the subfamily Helicinae, consistent with previous taxonomic studies (Korábek et al. 2019; Doğan et al. 2020; Kosicka et al. 2022). The close clustering of the families Hygromiidae and Geomitridae received strong nodal support (PP/BP = 1/100), corroborating the phylogenetic findings of Sei et al. (2017) and Kosicka et al. (2022), and further validating the sister‐group relationship between these taxa (Razkin et al. 2015).

In contrast, the family Xanthonychidae formed a sister group to Helicidae, a finding inconsistent with Zhao et al. (2023). Their ML tree, on the basis of amino acid sequence analysis, placed Xanthonychidae as the sister group to all other Helicoidea, whereas their BI analysis recovered Xanthonychidae as the sister group to the (Geomitridae + Helicidae) clade. The phylogenetic placement of Polygyridae within Helicoidea has varied across studies. One topology positions Polygyridae at the base of Helicoidea. Other analyses resolve Polygyridae and Camaenidae as sister groups (Minton et al. 2016; Yang et al. 2019; Guzmán et al. 2021; Kosicka et al. 2022; Zhao et al. 2023). Our results support the latter topology, albeit with only moderate nodal support (PP/BS = 0.94/50). These discrepancies may stem from differences in taxon sampling, datasets used, or analytical methods.

Notably, *Satsuma myomphala*, traditionally classified within Bradybaeninae, formed an independent lineage not monophyletic with other members of the subfamily. This finding supports the arguments by Nordsieck (2017) and Zhang et al. (2024) that the taxonomic status of this genus requires re‐evaluation. Nordsieck (2017) indicated that Satsuma is taxonomically distinct from other Bradybaena species. The Bayesian tree reconstructed by Sei et al. (2017) revealed Satsuma as sister to a clade containing Camaenidae, Bradybaenidae, and Polygyridae. Furthermore, Zhang et al. (2024) confirmed that *Satsuma* sensu lato (including its subgenus Coniglobus) forms a monophyletic group clearly distinct from lineages of Bradybaeninae and Hadrinae. They concluded that *Satsuma* and its original subgenera (Coniglobus, Luchuhadra, and Satsuma sensu stricto) should be considered a distinct subfamily.

This study, on the basis of mitochondrial genomic data, elucidates the major evolutionary framework of the superfamily Helicoidea. Although phylogenetic signals supporting these specific relationships exist, their strength within the mitochondrial genome dataset is not overwhelmingly dominant. The weak support for some nodes suggests they should be regarded as reasonable hypotheses warranting further validation rather than established conclusions. To unambiguously resolve these relationships, future studies should prioritize expanding taxon sampling (particularly for key underrepresented groups) and integrating multiple nuclear genetic markers for analysis. Such additional data will provide stronger phylogenetic signals, helping to determine whether current inconsistencies are due to stochastic error, model inadequacy, or genuine biological phenomena like incomplete lineage sorting.

## Author Contributions

**Xing‐Ming Ran:** conceptualization (equal), data curation (lead), investigation (equal), methodology (lead), visualization (lead), writing – original draft (lead), writing – review and editing (lead). **Hai‐Yong He:** funding acquisition (supporting), investigation (equal), resources (lead). **Lin Yang:** investigation (equal), project administration (lead). **Jian‐Kun Long:** investigation (equal). **Zhi‐Min Chang:** investigation (equal). **Zhong Li:** investigation (equal). **Junyi Gao:** investigation (equal). **Xiang‐Sheng Chen:** conceptualization (equal), funding acquisition (lead), methodology (supporting), supervision (lead), writing – review and editing (lead).

## Conflicts of Interest

The authors declare no conflicts of interest.

## Supporting information


**Appendix S1:** ece372282‐sup‐0001‐AppendixS1.zip.

## Data Availability

The mitochondrial genomes of newly generated in this study have been deposited in GenBank. A. ravida (GenBank: PQ166711), B. similaris (GenBank: PQ180368), and T. submissa (GenBank: PQ180369).
