# Supplementary material for: Mitochondrial Genomes of Three Species of the Family Camaenidae (Gastropoda: Stylommatophora): Structural Features, Codon Usage Patterns, and Phylogenetic Implications
Source: Ecol Evol. 2025 Oct 12;15(10):e72282. doi: 10.1002/ece3.72282 (PMC12515985; doi:10.1002/ece3.72282)
Supplement: Supplementary file 1 — Appendix S1: ece372282‐sup‐0001‐AppendixS1.zip. [file ECE3-15-e72282-s001.zip › Supinfo.docx]

**Appendix**

| **Table S1.** Summary of Mitogenomes used in analyses | | | |
| --- | --- | --- | --- |
| **Family** | **Species** | **Accession number** | **length** |
| Achatinidae | *Lissachatina fulica* | NC_024601.1 | 15057 |
| Arionidae | *Arion vulgaris* | NC_046044.1 | 14548 |
| Camaenidae | *Acusta ravida* | PQ166711.1 | 14135 |
| Camaenidae | *Aegista aubryana* | NC_029419.1 | 14238 |
| Camaenidae | *Aegista diversifamilia* | NC_027584.1 | 14039 |
| Camaenidae | *Bradybaena similaris* | PQ180368.1 | 14429 |
| Camaenidae | *Camaena cicatricosa* | NC_025511.1 | 13843 |
| Camaenidae | *Camaena poyuensis* | KT001074.1 | 13798 |
| Camaenidae | *Cochlostyla marinduquensis* | NC_071158.1 | 14806 |
| Camaenidae | *Dolicheulota formosensis* | NC_027493.1 | 14237 |
| Camaenidae | *Laeocathaica amdoana* | NC_070212.1 | 14660 |
| Camaenidae | *Mastigeulota kiangsinensis* | NC_024935.1 | 14029 |
| Camaenidae | *Satsuma myomphala* | NC_086491.1 | 14686 |
| Camaenidae | *Trichobradybaena submissa* | PQ180369.1 | 15471 |
| Geomitridae | *Helicella itala* | KT696546.1 | 13967 |
| Helicidae | *Cepaea hortensis* | OP910118.1 | 13988 |
| Helicidae | *Cepaea nemoralis* | U23045.1 | 14100 |
| Helicidae | *Cornu aspersum* | JQ417194.1 | 14050 |
| Helicidae | *Cylindrus obtusus* | NC_017872.1 | 14610 |
| Helicidae | *Helix lutescens* | OR684515.1 | 14066 |
| Helicidae | *Helix pelagonesica* | PQ213602.1 | 14004 |
| Helicidae | *Helix pomatia* | MK488031.1 | 14070 |
| Helicidae | *Helix pomatia* | NC_041247.1 | 14072 |
| Helicidae | *Helix thessalica* | OR684514.1 | 14069 |
| Xanthonychidae | *Micrarionta opuntia* | NC_060988.1 | 13971 |
| Hygromiidae | *Monacha cartusiana* | NC_072058.1 | 13894 |
| Philomycidae | *Meghimatium bilineatum* | NC_035429.1 | 13972 |
| Polygyridae | *Polygyra cereolus* | NC_032036.1 | 14008 |
| Polygyridae | *Praticolella mexicana* | NC_032079.1 | 14153 |

| **Table S2.** correlation coefficient between GC3 and GC12 | | | | | | |  |
| --- | --- | --- | --- | --- | --- | --- | --- |
| NAD1 |  | 0.584**^*^** |  | ATP8 |  | 0.664^*^ | |
| NAD2 |  | 0.813**^**^** |  | ATP6 |  | 0.811****** | |
| NAD3 |  | 0.844**^**^** |  | COX1 |  | 0.479 | |
| NAD4 |  | 0.790**^**^** |  | COX2 |  | 0.671^*^ | |
| NAD4L |  | 0.845**^**^** |  | COX3 |  | 0.489 | |
| NAD5 |  | 0.921**^**^** |  | CYTB |  | 0.634^*^ | |
| NAD6 |  | 0.441 |  | ******P<0.01 |  | *****P<0.05 | |


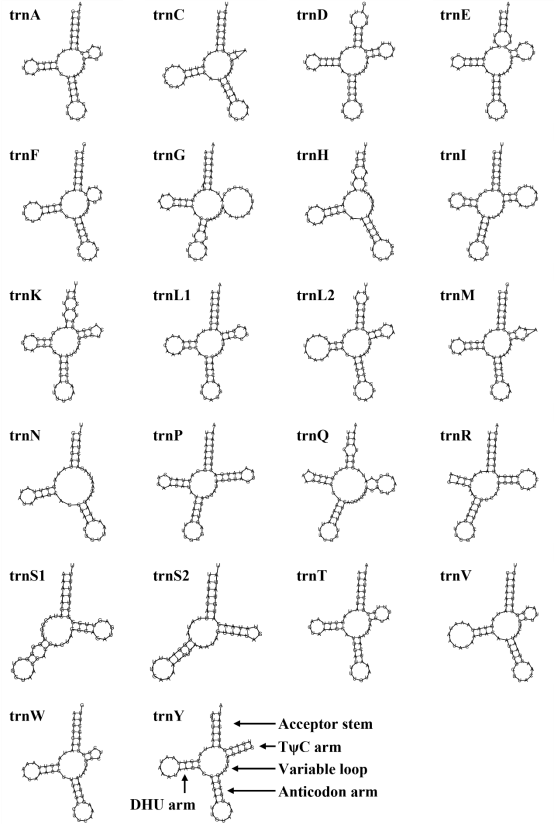


**Figure S1.** Putative secondary structure of the 22 tRNA genes identified in the mitogenome of *A. ravida*


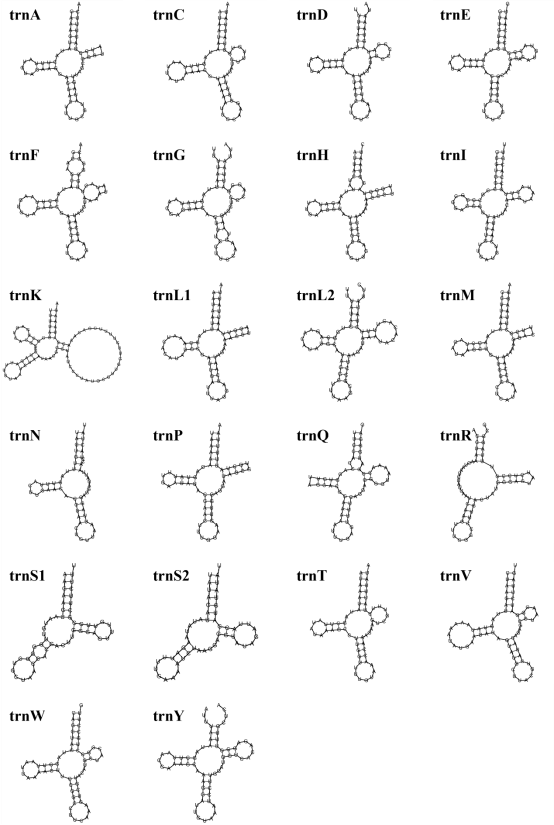


**Figure S2.** Putative secondary structure of the 22 tRNA genes identified in the mitogenome of *B. similaris*


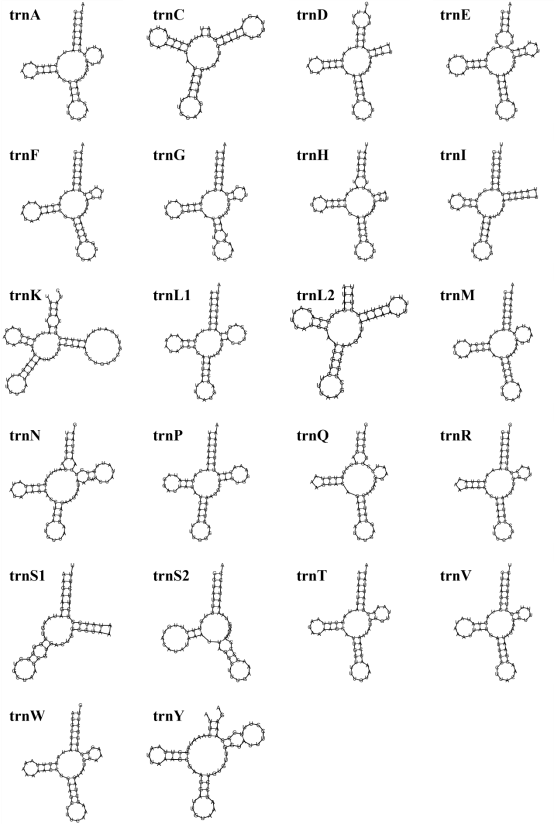


**Figure S3.** Putative secondary structure of the 22 tRNA genes identified in the mitogenome of *T. submissa*
